# Constipation

**Published:** 1874-12

**Authors:** 


### CONSTIPATION.

In spite of grapes and fruits and coarse food some people will, at times, suffer from a constipated condition of the bowels. In such a case try nux vomica. Buy half an ounce of the ordinary tincture of some good druggist, and put two drops of this tincture into a half an ounce of cold water in a phial, and keep it for use. Each day put one drop of this mixture in half a tumbler of cold water, and drink it. Repeat this operation for five or six times in a day; continue this process day after day, and in many cases a cure will result. Oatmeal mush, uubolted wheat bread, thoroughly boiled beans, and well cooked vegetables, are invaluable in constipation, and this diet would cure many cases; but these articles cannot always be had, and the appetite rebels against them in many instances; while the remedy which we propose above can be tried by anybody for five cents, and will be found beneficial in all cases which depend upon a lack of muscular power.

